# Effect of Novel Melanocortin Type 2 Receptor Antagonists on the Corticosterone Response to ACTH in the Neonatal Rat Adrenal Gland *In Vivo* and *In Vitro*

**DOI:** 10.3389/fendo.2016.00023

**Published:** 2016-03-21

**Authors:** Nasha K. Nensey, Jonathan Bodager, Ashley L. Gehrand, Hershel Raff

**Affiliations:** ^1^Department of Medicine, Medical College of Wisconsin, Milwaukee, WI, USA; ^2^Endocrine Research Laboratory, Aurora St. Luke’s Medical Center, Aurora Research Institute, Milwaukee, WI, USA

**Keywords:** ACTH, MC2R, corticosterone, adrenal cortex, antagonist

## Abstract

Stress-induced increases in neonatal corticosterone demonstrate a unique shift from ACTH independence to ACTH dependence between postnatal day 2 (PD2) and day 8 (PD8) in newborn rats. This shift could be due to the binding of a bioactive, non-­immunoreactive plasma ligand to the adrenocortical melanocortin 2 receptor (MC2R) (ACTH receptor). A potent MC2R antagonist would be useful to evaluate this phenomenon in the neonate. Therefore, we investigated the acute corticosterone response to ACTH_(1–39)_ injection in rat pups pretreated with newly developed MC2R antagonists (GPS1573 and GPS1574), which have not been tested *in vivo*. The doses used *in vivo* were based on their *in vitro* potency, with GP1573 being more potent than GPS1574. GPS1573 (PD2 and PD8), GPS1574 (PD2 only), or vehicle were injected intraperitoneally (ip) 10 min before baseline sampling. Then, 0.001 mg/kg of ACTH_(1–39)_ was injected ip, and subsequent blood samples obtained for the measurement of plasma corticosterone. Pretreatment of PD2 pups with GPS1573 demonstrated augmentation, rather than inhibition, of the corticosterone response to ACTH. In PD8 pups, pretreatment with 0.1 mg/kg GPS1573, but not 4 mg/kg, augmented the corticosterone response to ACTH. Pretreatment with GPS1574 attenuated the plasma corticosterone response to ACTH at 30 min in PD2 pups. The activity of these two compounds *in vivo* do not match their potency *in vitro*, with GPS1573 leading to a small augmentation of the corticosterone response to ACTH *in vivo* while GPS1574 resulted in inhibition.

## Introduction

Development of the neonatal hypothalamic–pituitary–adrenal (HPA) axis is critical for normal maturation of the lung, closing of the patent ductus arteriosus, and improving vasoconstrictor responses to catecholamines, as well as for stress-induced increases in blood glucose and blood pressure (1–3). In light of the increasing rate of premature births in the United States, it is important to understand the mechanisms of steroidogenesis and HPA axis maturation in the premature and full-term neonate (4). The neonatal rat model of human prematurity serves as a useful tool in studying the development of the HPA axis because the rat is an altricial animal. The full-term, newborn rat is immature compared to a full-term human neonate (5, 6). The primary glucocorticoid in the neonatal rat, corticosterone, is secreted from the zona fasciculata of the adrenal cortex in response to ACTH binding and activation of the melanocortin 2 receptor (MC2R) (7, 8). The binding of ACTH to MC2R leads to an increase in intracellular cyclic adenosine monophosphate (cAMP), causing activation of protein kinase A (PKA), and subsequent increase in movement of free cholesterol across the mitochondrial membrane into the cytosol (7–10). This transport of free cholesterol, mediated by the steroidogenic acute regulatory protein (StAR), is the rate-limiting step of steroidogenesis (11).

We have previously shown that on postnatal day 2 (PD2), rat pups exposed to hypoxic stress demonstrate an increase in corticosterone without an appreciable increase in immunoassayable ACTH and adrenal cAMP (12, 13). This phenomenon could be due to larger posttranslational products of POMC activating the MC2R receptor (14), which are not necessarily detected in our ACTH immunoassay. Although difficult to study without completely eliminating potential confounders, there are data suggesting that premature infants born at <32 weeks can mount a cortisol response without the large increase in plasma ACTH found in infants born >32 weeks gestational age (15). As stated earlier, the newborn rat is useful as a model of human prematurity (5, 6).

By postnatal day 8 (PD8), pups show an increase in corticosterone with the classic increase in immunoassayable ACTH and adrenal cAMP (12, 13, 16). The ability of the PD2 adrenal gland to respond to stress without a detectable increase in immunoreactive ACTH could be due to a bioactive, non-immunoreactive form of ACTH or another POMC product that can bind to and activate the adrenocortical MC2R. If the corticosterone response to stress in PD2 pups can be blocked by antagonizing the MC2R, it would suggest that a bioactive form of ACTH (not measured by immunoassay) is working through the MC2R in PD2 pups.

GPS1573 (Nle-P-f-R-w-F-K-A-V-G-K-K-R-R NH_2_) and GPS1574 [Nle-(E-f-R-w-F-K)-A-V-G-K-K-R-R NH_2_] are newly described, potent (IC_50_ = 66 ± 23 and 260 ± 1 nM, respectively), and dose-dependent antagonists of ACTH-stimulated MC2R activity *in vitro* (17). Note that the structures of the two compounds are similar except that GPS1574 has a ring structure. However, they have not been studied *in vivo*. The primary goal of the present study is to investigate the effect of GPS1573 and GPS1574 *in vitro* (adrenal cells) and *in vivo* (neonatal rats) in order to set the stage for its use in evaluating the role of endogenous ACTH in the neonatal adrenal stress response. We hypothesize that these MC2R antagonists are effective in adrenal cells from neonatal rats *in vitro*. Furthermore, we hypothesize that in the neonatal rat, GPS1573 and GPS1574, when given at a 100- to 4000-fold higher dose and 4000- to 8000-fold higher dose, respectively, than exogenous ACTH_(1–39)_, will attenuate the adrenocortical corticosterone response to ACTH.

## Materials and Methods

### Animal Treatment and Experimental Protocol

The animal protocol was approved by the Institutional Animal Care and Use Committee of Aurora Health Care. Timed-pregnant Sprague-Dawley rats at 14–17 gestational days (*N* = 45) were obtained from Harlan Sprague Dawley (Indianapolis, IN, USA), maintained on a standard diet, and had water available *ad libitum* in a controlled environment (0600–1800 hours lights on). Dams were allowed to deliver and care for their pups without interference until experimentation. The MC2R antagonists GPS1573 and GPS1574 were synthesized by Genepep (St. Jean de Védas, France) and reconstituted, as described previously (17).

### Adrenal Cell Preparation and GPS1573 and GPS1574 *In Vitro*

Adrenal cells from rats (*N* = 11 adults, 46 PD2 pups, and 26 PD8 pups) were dispersed and studied as previously described (18, 19). Briefly, rats were killed by decapitation, and both adrenal glands were removed and cleaned of surrounding fat. Only adult adrenal glands were decapsulated prior to cell dispersion. After a 90-min type I collagenase treatment and washing, viable cells were plated in a 96-well microtiter plate (10,000 cells/well) and pretreated for 1 h with (a) no antagonist (b) 750 nM GPS1573, or (c) 750-nM GPS1574 at 37°C in 10% CO_2_ (balance room air). After pretreatment, rat ACTH_(1–39)_ (Bachem) was added to cell suspensions at appropriate concentrations and incubated for 1 h. Medium was removed and immediately assayed for corticosterone (20).

### GPS1573 and GPS1574 Studies in the Neonatal Rat *In Vivo*

Rat pups were randomly assigned to experimental groups on the morning of the experiment. Then, rat pups were removed from the dams and placed in a small cage with adequate bedding, where they were allowed free range of motion and room to huddle. A variable control heating pad (Moore Medical, Farmington, CT, USA) was placed beneath the bedding and kept at the lowest setting required to maintain body temperature in normoxic rats at these ages (13, 21). After 10 min, rat pups were removed from the cage and quickly weighed.

In the GPS1573 studies, pups (both sexes; *N* = 252) were studied at PD2 (*N* = 96) and PD8 (*N* = 156) because these ages span the critical time during which the neonatal rat’s adrenal response to hypoxia shifts from immunoreactive ACTH independent to ACTH dependent (13, 16). Pups were injected intraperitoneally (ip) with either vehicle (10 μl/kg body wt of isotonic saline) or GPS1573 (diluted in isotonic saline) in low or high dose (0.1 or 4.0 mg/kg body wt, respectively). We chose to give GPS1573 ip because (a) it is a small peptide amenable to proper absorption by this route, (b) ACTH injections given ip are effective, and (c) subcutaneous injection was ineffective (data not shown).

In the GPS1574 studies, pups (both sexes, *N* = 42) were only studied at PD2, with the 60-min time point omitted because of drug’s limited supply, the data from the 60-min time point with GPS1573 described above, higher necessary dose based on the *in vitro* studies [current experiments and Ref. (17)], and its expense. GPS1574 was given at a dose of 4 or 8 mg/kg ip. Otherwise, the experiments were performed as described for GPS1573.

Ten minutes after GPS1573 or GPS1574 injection, a subset of pups was quickly decapitated and trunk blood was collected (baseline, time 0). Immediately after the baseline collection, 1 μg/kg (0.001 mg/kg) of porcine ACTH (Sigma Chemical, St. Louis, MO, USA) was injected ip, as described (22–24). Subsets of pups were decapitated at 15, 30, or 60 min (GPS1573 only) post-injection. In another group of pups (*N* = 163), the vehicle for ACTH injection (isotonic saline) was injected ip 10 min after GPS1573 or GPS1574 administration, and blood collected as described above. Trunk blood was collected in EDTA tubes (1 pup/sample), processed to plasma, and stored frozen (−20°C). Plasma corticosterone was measured by immunoassay, as described previously (MP Biomedicals, Orangeburg, NJ, USA) (25).

### Statistical Analyses

Corticosterone data were analyzed by two-way ANOVA. *Post hoc* analysis was performed by Holm–Sidak multiple range test (*P* < 0.05) (SigmaPlot 11.0). Data are presented as mean ± SEM.

## Results

### GPS1573 and GPS1574 *In Vitro*

**Figure 1:**
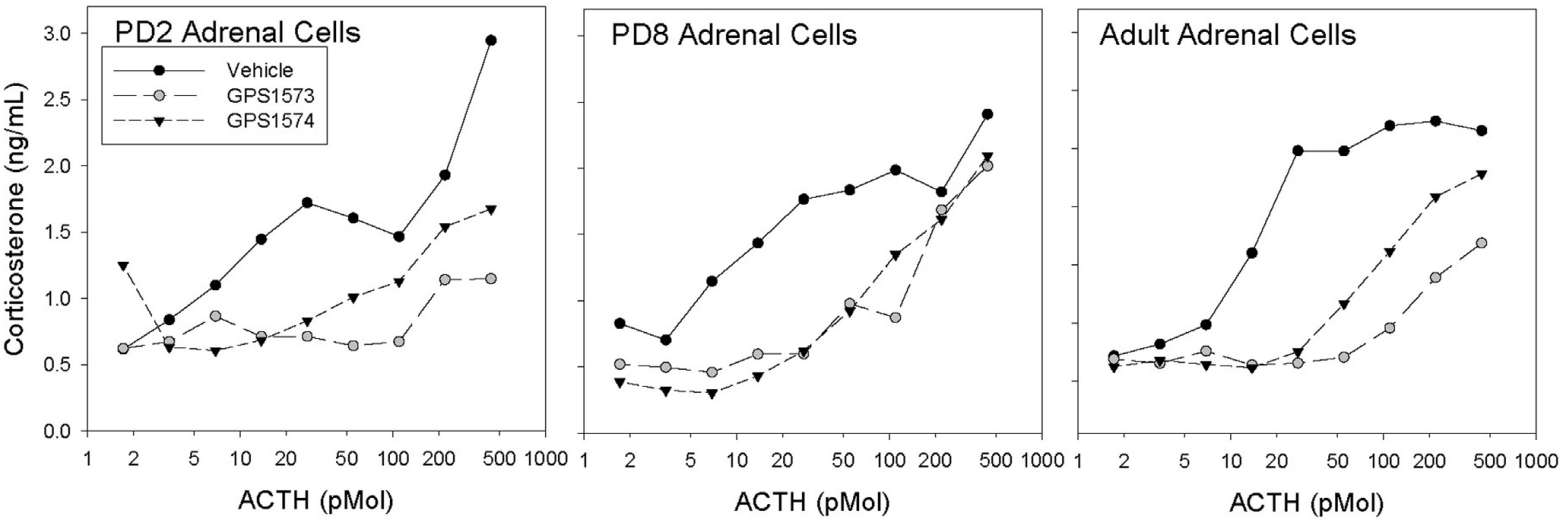
**Corticosterone responses to ACTH *in vitro* in adrenal cells from rats of different ages**. Cells were pretreated with vehicle, GPS1573, or GPS1574 (750 nM). Each point is the mean of two replicates.

Figure 1 shows the corticosterone response to ACTH in dispersed adrenal cells of rat pups (PD2 and PD8) and adults treated with GPS1573 and GPS1574. The cells from adult adrenal glands showed significant inhibition of the corticosterone response to ACTH *in vitro*, with GPS1573 being more potent than GPS1574. A similar response was observed in PD2 pup adrenal cells, as previously described for adult rats (17). In PD8 pup adrenal cells, however, there was equivalent inhibition between GPS1573 and GPS1574, compared to vehicle control.

### GPS1573 *In Vivo*

**Figure 2:**
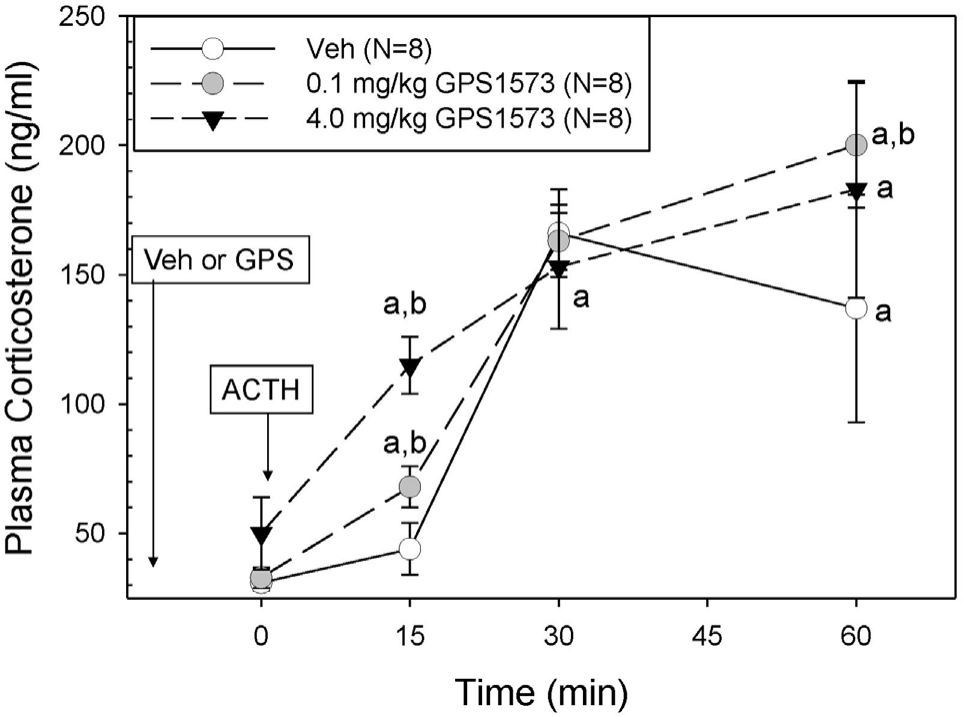
**Plasma corticosterone response to ACTH injection in postnatal 2 (PD2) rats pretreated with the low and high doses of GPS1573**. PD2 pups were injected with vehicle and low or high dose of GPS1573 (0.1 or 4.0 mg/kg body wt ip, respectively). Ten minutes later, baseline samples were obtained, and then porcine ACTH (0.001 mg/kg) was injected ip. ^a^Significantly different from baseline (0 min). ^b^Significantly different from vehicle (*P* < 0.05). Data are presented as means ± SE. *N* values (number of pups) for each mean are shown in the figure label.

The plasma corticosterone responses to exogenous ACTH in PD2 pups pretreated with either vehicle (for GPS1573) or the low or high dose of GPS1573 are shown in Figure 2. Baseline plasma corticosterone responses (10 min after injection of GPS1573) ranged from 30.4 ± 3.7 to 45.7 ± 8.7 ng/ml and were not different from our previously published baseline data without an injection 10 min before sampling (23, 24). PD2 pups pretreated with vehicle did not show an increase in corticosterone in response to ACTH at 15 min but did have an increase in corticosterone at 30 min (166.0 ± 17.1 ng/ml) and 60 min (137.2 ± 43.5 ng/ml). After pretreatment with the low dose of GPS1573, compared to vehicle, the PD2 pups showed a significantly augmented plasma corticosterone response at 15 min (67.8 ± 7.9 ng/ml) and 60 min (200.0 ± 23.6 ng/ml). When pretreated with the high dose of GPS1573, PD2 pups demonstrate an even greater augmentation of the corticosterone response at 15 min (115.5 ± 11.0 ng/ml), when compared to pretreatment with low dose or vehicle.

**Figure 3:**
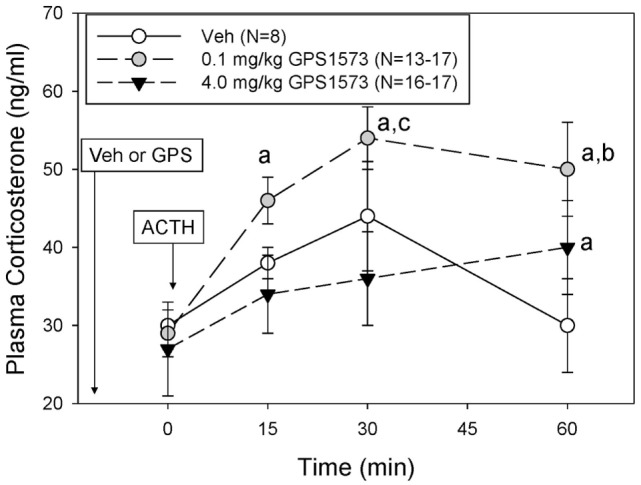
**Plasma corticosterone response to ACTH injection in postnatal 8 (PD8) rats pretreated with the low and high doses of GPS1573**. PD8 pups were injected with vehicle and low or high dose of GPS1573 (0.1 or 4.0 mg/kg body wt ip, respectively). Ten minutes later, baseline samples were obtained, and then porcine ACTH (0.001 mg/kg) was injected ip. ^a^Significantly different from baseline (0 min). ^b^Significantly different from vehicle (*P* < 0.05). ^c^Significantly different from 4.0 mg/kg. Data are presented as means ± SE. Note the *y*-axis range is lower than Figure 2 (PD2 pups). *N* values (number of pups) for each mean are shown in the figure label.

The plasma corticosterone responses to exogenous ACTH in PD8 pups pretreated with either vehicle (for GPS1573) or the low or high dose of GPS1573 are shown in Figure 3. Baseline plasma corticosterone responses (10 min after injection of GPS1573) ranged from 22.8 ± 3.7 to 25.7 ± 2.9 ng/ml and were not different from our previously published baseline data, without an injection 10 min before sampling (23, 24). The plasma corticosterone response to ACTH in PD8 pups was less than PD2 pups. In fact, there was no significant increase in corticosterone in response to ACTH in PD8 pups. Compared to vehicle, PD8 pups pretreated with the low dose of GPS1573 showed an augmentation of the plasma corticosterone response to ACTH at 15 min (45.8 ± 2.6 ng/ml), 30 min (54.5 ± 3.7 ng/ml), and 60 min (50.4 ± 6.5 ng/ml). The corticosterone response to ACTH in PD8 pups pretreated with the high dose of GPS1573 was not different than vehicle at 15 min (34.3 ± 4.5 ng/ml) and 30 min (36.0 ± 6.0 ng/ml) but was less than the low dose of GPS1573 at 60 min (40.4 ± 5.9 ng/ml).

We also evaluated a time control in which pups were pretreated with GPS1573 and then injected with vehicle for ACTH (Table 1). For the most part, there were no statistically significant changes in plasma corticosterone. It is important to note that baseline plasma corticosterone concentrations were similar to those in our prior studies without ip injection prior to baseline (23, 24). However, there was a small increase in plasma corticosterone at 15 min in PD2 pups pretreated with the high dose of GPS1573 and at 30 min in PD8 pups pretreated with the low dose of GPS1573.

**Table 1:** **Plasma corticosterone (nanogram per milliliter) response to vehicle (for ACTH injections) after pretreatment of PD2 and PD8 pups with the low dose (0.1 μg/kg) or high dose (4 μg/kg) of GPS1573**.

Age	Dose	Time (min)			
		Baseline	15	30	60
PD2	Low (*N* = 5–6)	25.7 ± 7.0	49.4 ± 16.6	37.1 ± 10.3	42.0 ± 7.7
	High (*N* = 7)	41.0 ± 9.6	67.4 ± 6.2^a^	25.4 ± 4.9	29.0 ± 9.4
PD8	Low (*N* = 5)	17.0 ± 4.1	24.6 ± 3.4	41.2 ± 4.4^a^^,^^b^	30.3 ± 11.5
	High (*N* = 11–12)	16.4 ± 2.6	21.0 ± 3.2	14.5 ± 3.2	15.7 ± 2.9

*^a^Different from baseline*.

*^b^Different from high dose within PD8*.

### GPS1574 *In Vivo*

**Figure 4:**
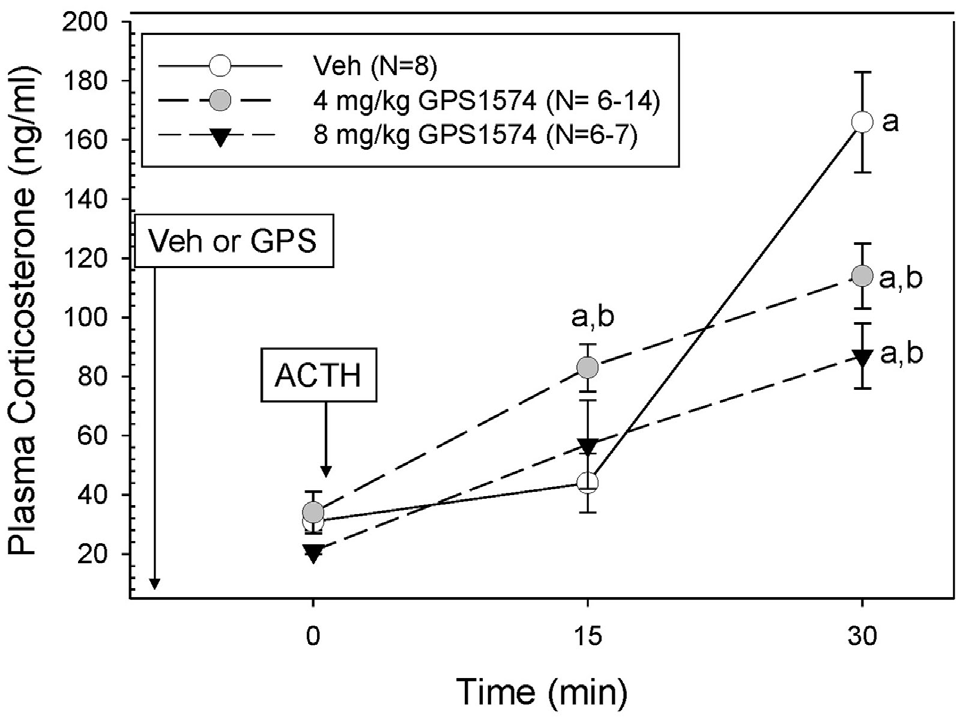
**Plasma corticosterone response to ACTH injection in postnatal 2 (PD2) rats pretreated with GPS1574**. Pups were injected with vehicle or GPS1574 (4 or 8 mg/kg body wt ip, respectively). Ten minutes later, baseline samples were obtained, and then porcine ACTH (0.001 mg/kg) was injected ip. ^a^Significantly different from baseline (0 min). ^b^Significantly different from vehicle (*P* < 0.05). Data are presented as means ± SE. *N* values (number of pups) for each mean are shown in the figure label.

Although we originally planned to only evaluate GPS1573 because of its higher potency *in vitro* (17), which was confirmed by our *in vitro* studies, the lack of inhibition with GPS1573 described above led us to do a limited number of new *in vivo* experiments with the apparently less potent GPS1574. We limited the GPS1574 study to PD2 (an age we are most interested in) through 30 min because of the need to use a higher dose of this very expensive drug, which was in limited supply and because the peak response to ACTH injection was at 30 min. As Figure 4 demonstrates, there was no inhibition at 15 min after ACTH administration, but a dose-dependent inhibition at 30 min was seen after ACTH injection. The time control for GPS1574 (saline injection rather than ACTH) did not reveal any changes in corticosterone (data not shown) and had baseline serum corticosterone concentrations similar to our previous studies without injection before baseline sampling.

## Discussion

This study evaluated the corticosterone response to exogenous ACTH injection in neonatal rats pretreated with potent, *in vitro* MC2R antagonists, which have not previously been tested *in vivo* (17). We hypothesized that these compounds would attenuate the adrenocortical MC2R, thereby resulting in a lower corticosterone response to ACTH in PD2 and PD8 rats. If these antagonists were effective *in vivo*, it would allow us to evaluate the possibility that stress-induced increases in corticosterone at PD2 that are independent of increases in immunoreactive plasma ACTH could be explained by binding of a non-immunoreactive corticotrophic ligand to the adrenocortical MC2R (12, 13, 16, 21). We chose to study neonatal rats based on previous studies (23, 24). Additionally, we are currently unable to study these compounds in adult rats because of the very large quantities of the GPS compounds needed, the compounds’ expense, and their limited supply.

Although the limited GPS1574 *in vivo* studies were performed after the responses to GPS1573 were ascertained, we will first discuss the GPS1574 data. GPS1574 resulted in an attenuated corticosterone response at 30 min after ACTH injection. We studied GPS1574 at a dose 4000- and 8000-fold higher than the exogenous ACTH dose. We were unable to use an even higher dose because of the limited supply of this compound. With the assumption that these GPS compounds are absorbed in a manner similar to ACTH, we calculated the plasma concentration of the GPS compounds to be at least 10- to 20-fold higher than the peak plasma ACTH concentration after injection. It is possible that these drugs are not as well absorbed as ACTH_(1–39)_ and/or that they are metabolized much more readily than ACTH_(1–39)_, perhaps requiring even higher doses ip. These preliminary results show promise for a more comprehensive analysis of this compound at much higher doses.

GPS1573, given *in vivo* at a 4000-fold higher dose of exogenous ACTH, did not antagonize the adrenal response to ACTH at either age. Rather, we demonstrated that pretreatment with GPS1573 augmented the corticosterone response to ACTH stimulation *in vivo*. In PD2 pups, the low dose of GPS1573 significantly augmented the corticosterone response to ACTH at 15 and 60 min compared to vehicle while the high dose demonstrated an even greater augmentation at 15 min compared to vehicle and low dose. In PD8 pups, pretreatment with the low dose of GPS1573 showed significant augmentation compared to vehicle at 60 min while pretreatment with the high dose showed no significant increase in corticosterone at any time point. GPS1573 did not consistently act as an agonist *per se*, with only small increases in corticosterone in response to ACTH vehicle (saline) at two time points.

This leads us to conclude that although GPS1573 acts as a competitive antagonist *in vitro* in adrenal cells from PD2, PD8, and adult cells, pretreatment with it *in vivo* results in an augmentation of the response to the natural ligand for the MC2R (ACTH). A similar phenomenon has been shown for nuclear (intracellular) receptors (26).

What could explain the effect demonstrated for a G-protein coupled receptor like the MC2R? First, it could be that the IC_50_ of GPS1573 is too high to be effective *in vivo*, despite the fact that we gave it at a 4000-fold higher dose than ACTH. The data from GPS1574 do not corroborate this notion since it has a higher IC_50_ *in vitro* but was effective *in vivo*. It is also possible that GPS1573 acts as an antagonist *in vitro* but is a biasing agonist *in vivo*. That is, *in vivo*, rather than blocking the receptor, it actually augments G-protein coupled transduction when ACTH binds to the MC2R (27, 28). This phenomenon could also be attributable to GPS1573 triggering a non-specific sympathetic nervous system-induced increase in the sensitivity of the adrenal cortex to ACTH (22, 29–36). Another possibility is that GPS1573 is inactivated shortly after being injected or that it has a very short half-life *in vivo*.

Potential drawbacks of our study design include the route of administration of GPS1573 and GPS1574. We chose ip administration *a priori*, since these antagonists are small compounds, and we have previously shown that ip ACTH injection is effective in stimulating corticosteronogenesis (22–24). We performed a few pilot studies with subcutaneous injection, which were ineffective (data not shown). However, an alternate route of administration (e.g., intravenous or intramuscular) may be more effective. It is also possible that these compounds would have been more effective *in vivo* in postpubertal rats. Alternate controllers of neonatal steroidogenesis, including postganglionic sympathetic input described above, have been proposed; however, the current study avoids these confounders by injecting ACTH rather than using stress as a stimulus to ACTH (22–24).

In conclusion, it appears that high dose of GPS1574 has potential as a competitive antagonist of ACTH *in vivo*. However, GPS1573 seems to act like a biasing agonist *in vivo* when given before an ACTH injection even though it is not consistently an agonist when given alone. The differences in behavior between these two compounds *in vivo* may be related to the ring structure of GPS1574.

## Author Contributions

NN, JB, AG, and HR all contributed substantially to the design and performance of the experiments and assays, writing and editing of the manuscript, approval of the final version, and agreement to account for all aspects of the work.

## Conflict of Interest Statement

The authors declare that the research was conducted in the absence of any commercial or financial relationships that could be construed as a potential conflict of interest.
